# Cost-Effectiveness of Metoclopramide, Paracetamol, and Ceftriaxone for the Prevention of Infections and Fever in Elderly Patients with Acute Stroke

**DOI:** 10.1177/23814683251386472

**Published:** 2026-01-17

**Authors:** Mathyn Vervaart, Jeroen C. de Jonge, Philip M. Bath, Hans Olav Melberg, Hendrik Reinink, Wouter M. Sluis, Lisa J. Woodhouse, H. Bart van der Worp, Anne Hege Aamodt, the PRECIOUS Investigators

**Affiliations:** Clinical Trial Unit, Oslo University Hospital, Oslo, Norway; Department of Neurology and Neurosurgery, Brain Center, University Medical Center Utrecht, Utrecht, Netherlands; Stroke Trials Unit, Mental Health & Clinical Neurosciences, School of Medicine, University of Nottingham, UK; Department of Community Medicine, The Arctic University of Norway, Tromsø, Norway; Clinical Trial Unit, Oslo University Hospital, Oslo, Norway; Department of Neurology and Neurosurgery, Brain Center, University Medical Center Utrecht, Utrecht, Netherlands; Department of Neurology and Neurosurgery, Brain Center, University Medical Center Utrecht, Utrecht, Netherlands; Stroke Trials Unit, Mental Health & Clinical Neurosciences, School of Medicine, University of Nottingham, UK; Department of Neurology and Neurosurgery, Brain Center, University Medical Center Utrecht, Utrecht, Netherlands; Department of Neurology, Oslo University Hospital, Oslo, Norway; Department of Neuromedicine and Movement Science, Norwegian University of Science and Technology, Trondheim, Norway

**Keywords:** cost-effectiveness, stroke, economic evaluation, decision modeling, value of information

## Abstract

**Objective.** Infections and fever after stroke are associated with poor functional outcome and death. We compared the cost-effectiveness of prophylactic metoclopramide, paracetamol, and ceftriaxone, either as monotherapies or in combination, with standard of care for reducing complications in older patients with acute stroke. **Methods.** We conducted a model-based analysis estimating expected lifetime costs, quality-adjusted life-years (QALYs), and net monetary benefit (NMB) from a Dutch health care perspective, given a willingness-to-pay threshold of 50,000 euros per QALY gained. The model synthesized patient-level data from the PREvention of Complications to Improve OUtcome in older patients with acute Stroke (PRECIOUS) trial with data from long-term observational studies. We conducted a value-of-information analysis to quantify the expected value of reducing decision uncertainty through additional data collection on key input parameters. **Results.** Combination therapy with metoclopramide, ceftriaxone, and paracetamol yielded the highest expected NMB and was the most cost-effective treatment option. When compared with standard care, this treatment option decreased costs by €6,438 and increased QALYs by 0.10, resulting in an incremental NMB of €11,721. The value-of-information analysis indicated that decision uncertainty was driven by uncertainty about the treatment effects on poststroke disability, measured by the modified Rankin Scale (mRS). Moreover, the analysis suggested that the expected value of reducing uncertainty through additional data collection on acute phase mRS scores far exceeds the expected costs. **Conclusion.** Our economic evaluation suggests that combination therapy with metoclopramide, paracetamol, and ceftriaxone may be the most cost-effective option for treating older patients with stroke in the Netherlands. However, there is likely great value in reducing uncertainty by conducting a new study that collects additional data on acute phase mRS scores.

**Highlights:**

- Prophylactic combination therapy with metoclopramide, paracetamol, and ceftriaxone may be the most cost-effective strategy for preventing infections and fever in elderly patients with acute stroke.
- There is substantial decision uncertainty, primarily driven by uncertainty regarding the treatment effects on functional outcome (modified Rankin Scale, mRS).
- Value-of-information analysis indicates that the value of reducing uncertainty through a new study on acute phase mRS scores is very high.

Stroke is the third leading cause of combined death and disability, leading to substantial costs for society.^1^ Infections and fever are common complications in the first days after stroke, especially in older patients and those with more severe stroke, and have been associated with increased risk of poor outcome and death.^2^ Evidence on the health and economic consequences of pharmacologic treatments to prevent or treat these complications and thereby to improve outcomes after stroke is, however, scarce.

The PREvention of Complications to Improve OUtcome in older patients with acute Stroke (PRECIOUS) randomized controlled trial assessed whether a pharmacologic strategy to prevent infections or fever with metoclopramide, ceftriaxone, paracetamol, or any combination of these would improve functional outcome in older patients with acute stroke.^3^ The primary outcome was the score on the modified Rankin Scale (mRS) at 90 d poststroke. The mRS is a 7-point scale, ranging from 0 (no disabilities) to 6 (death), designed to quantify the range of disability after a stroke.^4,5^ Within the PRECIOUS trial, the percentage of patients achieving a good functional outcome,^6^ defined as an mRS score of 0 to 2, ranged from 22.8% to 31.9% across different treatment combinations, and mortality rates varied from 18.7% to 24.8%, as detailed in the supplemental material. These variations in short-term clinical outcomes observed in the PRECIOUS trial could have important implications for ongoing costs of stroke care and broader health outcomes, such as long-term survival and quality of life.

Economic evaluation aims to provide decision makers with estimates of the expected costs and health benefits of new treatments compared with standard care, which is essential for making informed decisions about their adoption in clinical practice.^7^ In economic evaluation, health benefits are typically measured in terms of quality-adjusted life-years (QALYs), which combine quantity of life (remaining life expectancy) and quality of life after receiving an intervention into a single measure that can be compared across different indications and interventions.^8^ To calculate QALYs, economic evaluations are often required to translate intermediate outcomes measured over a short period, such as mRS scores,^9–12^ into estimates of long-term survival and quality of life—final outcomes that matter most to patients.^7,13^ Economic evaluation can thereby help decision makers prioritize treatments that provide the greatest health benefits for the resources invested.

Here, we report a preplanned economic evaluation to assess the long-term costs, QALYs, and cost-effectiveness of metoclopramide, paracetamol, and ceftriaxone for reducing complications in older patients with acute stroke. Our economic evaluation is based on patient-level data from the PRECIOUS trial supplemented with long-term data from observational studies.

## Methods

We developed a decision model to evaluate the cost-effectiveness of metoclopramide, paracetamol, and ceftriaxone, either as monotherapies or in combination, compared with standard care, for reducing complications in older patients with acute stroke. Our report follows the CHEERS guidelines for health economic evaluations^14^ and CHEERS-VOI extension for value-of-information analyses.^15^ A completed CHEERS-VOI checklist is provided in Appendix A. All analyses were performed in R software (version 4.2.2).^16^

### Patient Population

In the PRECIOUS trial, 1,493 patients aged 66 y or older with acute ischemic stroke or intracerebral hemorrhage and a National Institutes of Health Stroke Scale score of ≥6 were enrolled. These patients represent the target population for our economic evaluation, characterized by an average age of 80 y, with females constituting 50% of the patients.^3^

### Treatment Strategies

In the Netherlands, standard care for acute stroke follows national and international protocols tailored to stroke type.^17^ Upon hospital arrival, all patients undergo immediate brain imaging with a plain computed tomography (CT) scan. For those with ischemic stroke, this is further complemented by CT perfusion and CT angiography of the arteries, starting from the aortic arch. Approximately 25% of patients with ischemic stroke receive intravenous thrombolysis with alteplase or tenecteplase, while about 10% undergo endovascular thrombectomy. Patients experiencing intracerebral hemorrhage are treated with rapid blood pressure reduction if the systolic pressure exceeds 150 mm Hg. For those with subarachnoid hemorrhage due to an intracranial aneurysm, surgical clipping or endovascular treatment is used. All acute stroke patients are admitted to a stroke unit to facilitate the prevention and management of complications and to initiate early rehabilitation. However, in cases of compromised hemodynamics or respiration, patients are admitted to an intensive care unit for specialized care.

Participants in the PRECIOUS trial were randomly assigned in a 2×2×2 factorial, multicenter design to receive 1 of 8 treatment strategies: metoclopramide (10 mg 3 times daily), ceftriaxone (2,000 mg once daily), and paracetamol (1,000 mg 4 times daily), either as monotherapies or in combination, or standard care only.^3^ Treatment was initiated within 24 h of symptom onset and continued for 4 d. We considered both monotherapies and combination therapies, as well as standard care, to enable joint decision making for the full set of 8 mutually exclusive and collectively exhaustive treatment options.^18^ The treatment effects in our economic evaluation were directly based on the observed distributions of mRS scores at 90 d poststroke from each of the 8 randomized treatment groups in the PRECIOUS trial.^3^

### Model Overview

**Figure 1:**
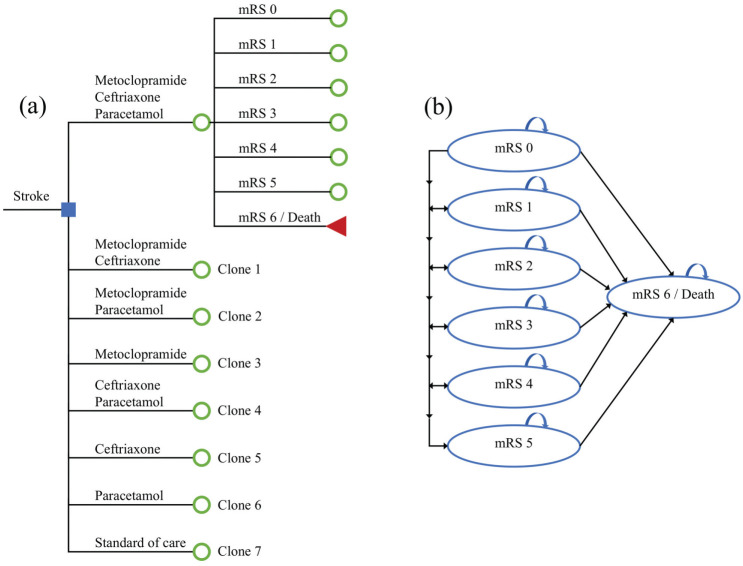
(a) Decision tree structure and (b) state-transition model structure. mRS, modified Rankin Scale.

We developed a decision model that consists of 2 components: a decision tree (Figure 1a) that captures the initial 90 d after stroke, and a state-transition model (Figure 1b) that simulates the long-term health and economic consequences after stroke.^13^ This 2-component model structure, along with the use of health states based on the mRS, is commonly employed in economic evaluations of stroke treatments.^9–12^ A cohort of 80-y-old patients enters the model on admission to the hospital for acute ischemic stroke and receives 1 of the following treatments: standard of care; monotherapy with metoclopramide, paracetamol, or ceftriaxone; or combination therapy. Subsequently, patients enter 1 of 7 possible health states according to the degree of disability as assessed by the mRS at 90 d poststroke. Treatment outcomes are assumed to occur during this 90-d acute phase, influencing the distribution of patients into the mRS-based health states. After the initial acute phase, the surviving patients transition into the long-term state-transition model to estimate expected costs and QALYs over a time horizon of 20 y until a maximum age of 100 y or death, whichever occurs first. During each model cycle, patients may remain in the same health state, experience a recurrent stroke and recover to the same mRS state or transition to a worse mRS state, or die from other causes. We used weekly model cycles to reduce the discrete-time approximation error.^19^

### Model Outcomes

Model outcomes included monetary costs (2024 euros) and QALYs for each of the 8 treatment strategies over a time horizon of 20 y. Since most patients enrolled in the PRECIOUS trial were from the Netherlands, we adopted a Dutch health care payer perspective. We discounted costs and QALYs at 3% and 1.5% per year, respectively, following the Dutch guidelines for economic evaluations in health care.^20^ A scenario analysis using a common discount rate of 3.5% for both costs and QALYs is presented in Appendix G. All costs were updated to 2024 euros using the medical component of the Dutch consumer price index obtained from Statistics Netherlands.^21^ To evaluate cost-effectiveness, we computed the expected net monetary benefit (NMB) for each treatment option, given by

(1) NMB=QALY×λ-Cost

where λ is a willingness-to-pay threshold for the incremental cost per QALY gained set by the decision maker. The most cost-effective treatment option is the one that provides the greatest expected NMB. We computed the NMB for willingness-to-pay thresholds of 20,000, 50,000 and 80,000 euros per QALY gained, corresponding to the range of possible thresholds used by the Dutch National Healthcare Institute.^22^ In addition to the NMB analysis, we constructed a cost-effectiveness frontier to determine which treatment options are cost-effective at various thresholds and identify those that are (extended) dominated. The frontier involves assessing incremental cost-effectiveness ratios (ICERs), defined as the incremental cost per QALY gained for treatments on the frontier, ranked from lowest to highest cost. To determine the most relevant willingness-to-pay threshold, we computed the proportional shortfall in the target population.^23^ Proportional shortfall quantifies disease severity by measuring the expected loss in QALYs over the patient’s remaining lifetime due to the disease, expressed as a proportion of the QALYs they would potentially have if the disease were absent. The Dutch Healthcare Institute assigns willingness-to-pay thresholds of 20,000, 50,000, or 80,000 euros per QALY gained for proportional shortfalls ranging from 0.10 to 0.40, 0.41 to 0.70, and 0.71 to 1.00, respectively.^22^

### Model Input Parameters

Patient-level data from the PRECIOUS trial were used to inform model input parameters related to treatment effects, quality-of-life weights, and acute phase resource use. Data from the literature provided inputs for unit costs, long-term care costs, as well as the long-term risks of stroke recurrence and mortality. Parameter values, distributions, and data sources are presented in Table 1.

**Table 1:** Model Parameters and Data Sources

Parameter Description	Mean	Distribution	Source
Acute phase mRS (0–6) probabilities			
$\begin{array}{c} \mathrm{SoC} \end{array}$	$\begin{array}{c} 0.07/0.07/0.09/0.21/0.19/0.12/0.24 \end{array}$	Dirichlet	PRECIOUS trial^3^
$\begin{array}{c} \mathrm{Par} \end{array}$	$\begin{array}{c} 0.02/0.10/0.10/0.25/0.19/0.15/0.19 \end{array}$	$\begin{array}{c} \mathrm{Dirichlet} \end{array}$	PRECIOUS trial^3^
$\begin{array}{c} \mathrm{Cef} \end{array}$	$\begin{array}{c} 0.06/0.09/0.17/0.15/0.22/0.12/0.19 \end{array}$	$\begin{array}{c} \mathrm{Dirichlet} \end{array}$	PRECIOUS trial^3^
$\begin{array}{c} \mathrm{Cef}-\mathrm{Par} \end{array}$	$\begin{array}{c} 0.05/0.07/0.09/0.19/0.19/0.13/0.28 \end{array}$	$\begin{array}{c} \mathrm{Dirichlet} \end{array}$	PRECIOUS trial^3^
$\begin{array}{c} \mathrm{Met} \end{array}$	$\begin{array}{c} 0.04/0.10/0.13/0.21/0.13/0.16/0.23 \end{array}$	$\begin{array}{c} \mathrm{Dirichlet} \end{array}$	PRECIOUS trial^3^
$\begin{array}{c} \mathrm{Met}-\mathrm{Par} \end{array}$	$\begin{array}{c} 0.05/0.11/0.13/0.18/0.19/0.14/0.20 \end{array}$	$\begin{array}{c} \mathrm{Dirichlet} \end{array}$	PRECIOUS trial^3^
$\begin{array}{c} \mathrm{Met}-\mathrm{Cef} \end{array}$	$\begin{array}{c} 0.03/0.12/0.14/0.21/0.14/0.16/0.20 \end{array}$	$\begin{array}{c} \mathrm{Dirichlet} \end{array}$	PRECIOUS trial^3^
$\begin{array}{c} \mathrm{Met}-\mathrm{Cef}-\mathrm{Par} \end{array}$	$\begin{array}{c} 0.07/0.10/0.14/0.13/0.17/0.14/0.25 \end{array}$	$\begin{array}{c} \mathrm{Dirichlet} \end{array}$	PRECIOUS trial^3^
Stroke recurrence rate			
$\begin{array}{c} \mathrm{Weibull}\;\log\;\mathrm{shape}\\ \mathrm{Weibull}\;\log\;\mathrm{scale} \end{array}$	$\begin{array}{c} -0.57\\ 5.49 \end{array}$	$\begin{array}{c} \mathrm{Bivariate}\\ \mathrm{Normal} \end{array}$	Skajaa et al.^24^
$\begin{array}{c} \mathrm{Recurrence}\;\mathrm{HR}\;\mathrm{mRS}\;0,\;1-2,\;3+ \end{array}$	$\begin{array}{c} 1/1.18/1.36 \end{array}$	$\begin{array}{c} \mathrm{Lognormal} \end{array}$	de Havenon et al.^25^
Mortality rate			
$\begin{array}{c} \mathrm{Mortality}\;\mathrm{rate}\;\mathrm{age}\;80-99\; \end{array}$	$\begin{array}{c} 0.001-0.008 \end{array}$	$\begin{array}{c} \mathrm{Constant} \end{array}$	Statistics Netherlands^26^
$\begin{array}{c} \mathrm{Mortality}\;\mathrm{HR}\;\mathrm{mRS}\;0 \end{array}$	$\begin{array}{c} 1.20 \end{array}$	$\begin{array}{c} \mathrm{Lognormal} \end{array}$	Shavelle et al.^27^
$\begin{array}{c} \mathrm{Mortality}\;\mathrm{HR}\;\mathrm{mRS}\;1-5 \end{array}$	$\begin{array}{c} 1.42/1.87/2.17/3.1/6.91 \end{array}$	$\begin{array}{c} \mathrm{Lognormal} \end{array}$	Huybrechts et al.^28^
Utility weights			
$\begin{array}{c} \mathrm{mRS}\;0-6 \end{array}$	$\begin{array}{c} 0.94/0.85/0.78/0.59/0.30/0.14/0.00 \end{array}$	$\begin{array}{c} \mathrm{Beta} \end{array}$	PRECIOUS trial^3^
Acute phase length of stay (days)			
$\begin{array}{c} \mathrm{Hospital},\;\mathrm{mRS}\;0-6 \end{array}$ Rehabilitation, mRS 0–6	$\begin{array}{c} 8.9/9.8/14.5/18.9/24.9/29.7/14.9 \end{array}$		PRECIOUS trial^3^
	$\begin{array}{c} 5.6/7.9/16.0/29.7/39.5/31.2/4.1 \end{array}$	$\begin{array}{c} \mathrm{Gamma} \end{array}$	PRECIOUS trial^3^
$\begin{array}{c} \mathrm{Nursing}\;\mathrm{home},\;\mathrm{mRS}\;0-6 \end{array}$	$\begin{array}{c} 0.0/0.7/0.6/2.4/9.0/16.0/3.4 \end{array}$	$\begin{array}{c} \mathrm{Gamma} \end{array}$	PRECIOUS trial^3^
$\begin{array}{c} \mathrm{Home},\;\mathrm{mRS}\;0-6 \end{array}$	$\begin{array}{c} 75.4/71.5/59.0/39.0/16.6/13.1/3.5 \end{array}$	$\begin{array}{c} \mathrm{Gamma} \end{array}$	PRECIOUS trial^3^
Acute phase procedures (proportion)			
$\begin{array}{c} \mathrm{Intravenous}\;\mathrm{thrombolysis},\;\mathrm{mRS}\;0-6 \end{array}$	$\begin{array}{c} 0.43/0.54/0.57/0.52/0.44/0.40/0.43 \end{array}$	$\begin{array}{c} \mathrm{Gamma} \end{array}$	PRECIOUS trial^3^
$\begin{array}{c} \mathrm{Mechanical}\;\mathrm{thrombectomy},\;\mathrm{mRS}\;0-6 \end{array}$	$\begin{array}{c} 0.20/0.30/0.30/0.24/0.29/0.28/0.23 \end{array}$	$\begin{array}{c} \mathrm{Gamma} \end{array}$	PRECIOUS trial^3^
$\begin{array}{c} \mathrm{Carotid}\;\mathrm{endarterectomy} \end{array}$	$\begin{array}{c} 0.06 \end{array}$	$\begin{array}{c} \mathrm{Beta} \end{array}$	Buisman et al.^29^
Acute phase unit costs (€)			
$\begin{array}{c} \mathrm{Met}\;\mathrm{drug}\;\mathrm{cost},\;\mathrm{per}\;\mathrm{patient} \end{array}$	$\begin{array}{c} 1.08 \end{array}$	$\begin{array}{c} \mathrm{Constant} \end{array}$	Dutch list prices^30^
$\begin{array}{c} \mathrm{Par}\;\mathrm{drug}\;\mathrm{cost},\;\mathrm{per}\;\mathrm{patient} \end{array}$	$\begin{array}{c} 1.60 \end{array}$	$\begin{array}{c} \mathrm{Constant} \end{array}$	Dutch list prices^30^
Cef drug cost, per patient	$\begin{array}{c} 74.72 \end{array}$	$\begin{array}{c} \mathrm{Constant} \end{array}$	Dutch list prices^30^
$\begin{array}{c} \mathrm{Hospital},\;\mathrm{per}\;\mathrm{day} \end{array}$	$\begin{array}{c} 683 \end{array}$	$\begin{array}{c} \mathrm{Gamma} \end{array}$	Dutch costing manual^31^
$\begin{array}{c} \mathrm{Rehabilitation}\;\mathrm{centre},\;\mathrm{per}\;\mathrm{day} \end{array}$	$\begin{array}{c} 379 \end{array}$	$\begin{array}{c} \mathrm{Gamma} \end{array}$	Dutch costing manual^31^
$\begin{array}{c} \mathrm{Nursing}\;\mathrm{home},\;\mathrm{per}\;\mathrm{day} \end{array}$	$\begin{array}{c} 308 \end{array}$	$\begin{array}{c} \mathrm{Gamma} \end{array}$	Dutch costing manual^31^
$\begin{array}{c} \mathrm{Visits},\;\mathrm{per}\;\mathrm{patient} \end{array}$	$\begin{array}{c} 250 \end{array}$	Gamma	Buisman et al.^29^
$\begin{array}{c} \mathrm{Diagnostics}\;\mathrm{and}\;\mathrm{imaging},\;\mathrm{per}\;\mathrm{patient} \end{array}$	$\begin{array}{c} 586 \end{array}$	$\begin{array}{c} \mathrm{Gamma} \end{array}$	Buisman et al.^29^
$\begin{array}{c} \mathrm{Allied}\;\mathrm{health}\;\mathrm{services},\;\mathrm{per}\;\mathrm{patient} \end{array}$	$\begin{array}{c} 257 \end{array}$	$\begin{array}{c} \mathrm{Gamma} \end{array}$	Buisman et al.^29^
$\begin{array}{c} \mathrm{Carotid}\;\mathrm{endarterectomy} \end{array}$	$\begin{array}{c} 5,426 \end{array}$	$\begin{array}{c} \mathrm{Gamma} \end{array}$	Buisman et al.^29^
$\begin{array}{c} \mathrm{Intravenous}\;\mathrm{thrombolysis} \end{array}$	$\begin{array}{c} 1,130 \end{array}$	$\begin{array}{c} \mathrm{Gamma} \end{array}$	van den Berg et al.^32^
$\begin{array}{c} \mathrm{Endovascular}\;\mathrm{thrombectomy} \end{array}$	$\begin{array}{c} 11,797 \end{array}$	$\begin{array}{c} \mathrm{Gamma} \end{array}$	van den Berg et al.^32^
$\begin{array}{c} \mathrm{Intravenous}\;\mathrm{infusion} \end{array}$	$\begin{array}{c} 180 \end{array}$	$\begin{array}{c} \mathrm{Gamma} \end{array}$	NICE TA650^33^
Long-term care costs, per week (€)			
$\begin{array}{c} \mathrm{mRS}\;0-6 \end{array}$	$\begin{array}{c} 127/127/183/412/925/1,208 \end{array}$	$\begin{array}{c} \mathrm{Gamma} \end{array}$	van Voorst et al.^34^
Indirect medical costs, per week (€)			
$\begin{array}{c} \mathrm{Age}-\mathrm{related}\;\mathrm{changes},\;\mathrm{age}\;80-99 \end{array}$	$\begin{array}{c} 0-704 \end{array}$	$\begin{array}{c} \mathrm{Constant} \end{array}$	PAID 3.0^35^

Cef, ceftriaxone; HR, hazard ratio; Met, metoclopramide; mRS, modified Rankin Scale; Par, paracetamol. All costs were converted to 2024 euros using the medical care component of the consumer price index.

### Transition Probabilities

The initial probabilities of entering specific mRS-based health states—reflecting the treatment effects—are derived from the observed distributions of mRS scores at 90 d poststroke for each of the 8 randomized treatment groups in the PRECIOUS trial.^3^ The long-term risk of stroke recurrence was estimated by calibrating a Weibull model to data from a Danish registry study on patients aged 70 to 79 y with ischemic stroke.^24^ The Weibull model reflects the decreasing risk of stroke recurrence over time (Figure C1 in the supplemental material). To incorporate the impact of mRS scores on recurrent stroke risk, we applied hazard ratios from the study by de Havenon et al.^25^ to adjust the scale parameter of the Weibull model. We normalized the adjusted scale parameters using the distribution of mRS under standard care to avoid overestimating the overall stroke recurrence risk. We used the initial probabilities associated with entering specific mRS health states under standard of care to approximate the probabilities of either remaining in the same mRS state or transitioning to a worse one following a recurrent stroke. This approach assumes that a recurrent stroke would have the same characteristics as the index stroke under standard of care, a conservative assumption supported by a previous cost-effectiveness study and registry data.^9,24^ We estimated mortality rates for mRS 0–5 at 90 d poststroke by applying hazard ratios from the literature^27,28^ to the age-specific mortality hazard from national life tables.^26^ Overall survival curves, conditional on patients surviving to 90 d poststroke and stratified by mRS, are presented in Figure C2 in the supplemental material.

### Costs

Using patient location data from the PRECIOUS trial during the 90 d of follow-up, we determined the length of stay during the acute phase, stratified by mRS health state, at the following locations: hospital, rehabilitation center, nursing home, and patients’ own homes. We sourced the unit costs per day for hospital, rehabilitation center, and nursing home stays from the Dutch costing manual for economic evaluations in health care (2024).^31^ We also used PRECIOUS trial data to estimate the probability of patients in each mRS health state receiving intravenous thrombolysis and mechanical thrombectomy during the acute phase. We derived the unit costs for these procedures from the Multicentre Randomized Clinical Trial of Endovascular Treatment for Acute Ischemic Stroke in the Netherlands (MR CLEAN).^32^ In addition, we obtained the procedural costs of carotid endarterectomy, as well as per-patient costs related to follow-up visits, diagnostic and imaging tests, and the use of allied health services during the acute phase following a stroke from the costing study by Buisman et al.,^29^ which evaluated hospital resource utilization and associated stroke costs in the Netherlands. Furthermore, the MR CLEAN trial provided the mRS-specific long-term care costs.^34^ We assumed that the daily costs for patients staying at home during the acute phase were equivalent to the daily long-term care costs. We incorporated age-related changes in indirect medical costs based on per capita health care expenditure data from the Dutch Practical Application to Include Disease Costs (PAID) tool (version 3.0).^35^

### Health-Related Quality of Life

We calculated utility weights that reflect the health-related quality of life experienced in the mRS-based health states. These utility weights, which can theoretically range from −0.446 (worse than death) to 1 (representing perfect health), were derived by applying Dutch tariffs^36^ to EQ-5D-5L^37^ data collected in the PRECIOUS trial at 90 d of follow-up. We computed QALYs by multiplying the time spent in the health states with the corresponding utility weights.^38^

### Analysis of Patient-Level Data

Patient-level data from the PRECIOUS trial were analyzed to inform model parameters as follows. The effectiveness estimates, represented by the distribution of mRS scores at 90 d poststroke for each treatment arm, were based on observed proportions within each arm of the PRECIOUS trial. Length-of-stay data for various care settings (hospital, rehabilitation center, nursing home, home) during the acute phase were derived from observed mean days across different mRS health states within the 90-d follow-up of the trial. Utility weights reflecting health-related quality of life were calculated based on mean EQ-5D-5L values within each mRS health state. In addition, the probabilities of patients in each mRS health state receiving intravenous thrombolysis and mechanical thrombectomy during the acute phase were estimated based on observed proportions in the trial.

A summary of missing data and complete case values for the abovementioned trial-informed parameters is presented in Appendix D of the supplemental material. Overall data completeness was as follows: 98.6% complete for mRS scores, 74.5% for EQ-5D scores, 86.5% for intravenous thrombolysis and mechanical thrombectomy, and 87.9% for institutional length of stay. We addressed missing data using multivariate imputation by chained equations.^39^ Additional details on the imputation process and a summary of the imputed values are available in Appendix E of the supplemental material.

### Probabilistic Analysis

We conducted a probabilistic analysis to assess the impact of joint parameter uncertainty on the cost-effectiveness estimates.^13^ This involved sampling 10,000 values from probability distributions that represent plausible ranges for each model parameter, based on patient-level data from the PRECIOUS trial and additional literature sources (detailed in Table B1 of the supplemental material). We constructed a cost-effectiveness scatterplot to illustrate the simulated costs and QALYs for the treatment options. To evaluate the probability of each treatment option being the most cost-effective, we constructed cost-effectiveness acceptability curves (CEACs) and a cost-effectiveness acceptability frontier (CEAF) for various willingness-to-pay thresholds.^40^

### Value-of-Information Analysis

We assessed the expected cost of decision uncertainty by calculating the expected value of perfect information (EVPI), which provides an upper bound on the potential value of conducting further research to reduce uncertainty.^41^ To understand which parameters most influence decision uncertainty, we computed the expected value of partial perfect information (EVPPI) for relevant groups of parameters using nonparametric regression.^42^ Specifically, we used Bayesian Additive Regression Trees (BART) as implemented in the voi R package,^43^ a method particularly suitable for evaluating joint EVPPI across many parameters, such as the 7-category mRS scores.

In addition, we estimated the expected value of sample information (EVSI), which quantifies the expected value to the decision maker of reducing uncertainty through a proposed data collection exercise.^41^ We computed the EVSI for a hypothetical new study that collects additional data on acute phase mRS scores for the 8 treatment strategies considered in the PRECIOUS trial. We calculated the EVSI using BART for study sample sizes of 160, 320, 800, and 4,000 patients, with 10,000 simulated data sets per sample size, assuming even allocation to the trial arms. We interpolated the EVSI estimates across the 4 sample sizes using an asymptotic regression model.^44^ We also calculated the expected net benefit of sampling (ENBS), which represents the net value of the hypothetical new study. The ENBS is the difference between the population-level EVSI and the cost of the hypothetical new study. To compute the population-level EVSI, we estimated a monthly incidence of 1,291 eligible patients. This was derived from national stroke statistics (an annual incidence of 41,300),^45^ which were adjusted to reflect the proportion of patients meeting the PRECIOUS trial’s eligibility criteria: approximately 75% for age (≥66 y)^46,47^ and 50% for stroke severity (National Institutes of Health Stroke Scale ≥6).^48^ We calculated the population-level EVSI for decision horizons of 60 to 240 mo, adjusted for an enrollment rate of 80 patients per month and follow-up period of 3 mo as outlined in the PRECIOUS trial protocol, as well as an analysis and reporting period of 6 mo. Trial costs included fixed costs of 4,6 million euros and variable costs of 500 euros per patient, derived from the PRECIOUS trial. We discounted the ENBS estimates at an annual rate of 3%.

## Results

### Cost-Effectiveness

The probabilistic cost-effectiveness results, including the mean cost, QALYs, and NMB for each treatment option, are reported in Table 2. At willingness-to-pay thresholds of 20,000, 50,000, and 80,000 euros per QALY gained, combination treatment with metoclopramide, ceftriaxone, and paracetamol yielded the highest expected NMB, offering the lowest expected costs of 137,656 euros while providing expected QALYs of 2.45. This combination therapy reduced costs by 6,438 euros and increased QALYs by 0.10 when compared with standard care. Ceftriaxone monotherapy, with an expected cost of 150,766 euros and expected QALYs of 2.59, was the most effective treatment strategy. When compared with standard care, ceftriaxone monotherapy increased costs by 6,672 euros and QALYs by 0.25. Compared with standard of care, all treatment options resulted in increased QALYs, with the exception of combination treatment with ceftriaxone and paracetamol.

**Table 2:** Probabilistic Cost-Effectiveness Results (Discounted)^a^

Strategy	Cost	QALYs	NMB, λ = 20,000	NMB, λ = 50,000	NMB, λ = 80,000
SoC	144,094 (7,173)	2.35 (0.15)	−97,122 (7,554)	−26,664 (9,945)	43,794 (13,447)
Par	153,119 (7,257)	2.38 (0.14)	−105,618 (7,756)	−34,366 (10,135)	36,887 (13,525)
Cef	150,766 (7,990)	2.59 (0.18)	−98,872 (8,877)	−21,030 (12,205)	56,812 (16,625)
Cef-Par	139,805 (8,534)	2.08 (0.18)	−98,175 (8,771)	−35,730 (11,498)	26,714 (15,697)
Met	139,335 (6,904)	2.41 (0.15)	−91,089 (7,348)	−18,720 (9,916)	53,649 (13,583)
Met-Par	147,056 (7,436)	2.50 (0.16)	−97,072 (8,048)	−22,095 (10,878)	52,881 (14,802)
Met-Cef	144,496 (7,994)	2.50 (0.19)	−94,483 (8,681)	−19,464 (12,042)	55,555 (16,663)
Met-Cef-Par	137,656 (8,583)	2.45 (0.21)	−**88,571 (9,318)**	−**14,943 (13,187)**	**58,684 (18,485)**

Cef, ceftriaxone; Met, metoclopramide; NMB, net monetary benefit; Par, paracetamol; QALY, quality-adjusted life-year; SE, standard error; SoC, standard of care; λ , willingness-to-pay threshold (incremental cost per QALY gained).

a All values are expressed as mean (standard error). The treatment option with the greatest net monetary benefit is displayed in bold. Costs and NMB are reported in euros.

**Figure 2:**
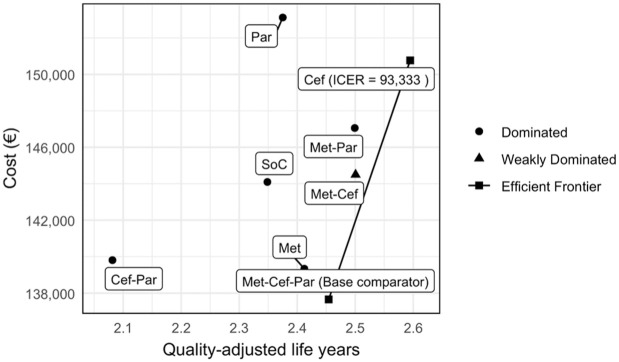
Cost-effectiveness frontier. Cef, ceftriaxone; ICER, incremental cost-effectiveness ratio; Met, metoclopramide; Par, paracetamol; SoC, standard of care.

A cost-effectiveness frontier is depicted in Figure 2. Only 2 treatments are on the efficient frontier: combination therapy with metoclopramide, paracetamol, and ceftriaxone, and ceftriaxone monotherapy. Combination therapy with metoclopramide, paracetamol, and ceftriaxone is the base cost-effective comparator as the lowest-cost strategy. Conversely, ceftriaxone monotherapy is the only other strategy on the efficient frontier, having an ICER of 93,333 euros per QALY gained compared with combination therapy. This indicates that ceftriaxone monotherapy is expected to be cost-effective at willingness-to-pay thresholds of 95,000 euros per QALY gained and higher.

### Proportional Shortfall

The undiscounted remaining QALYs for patients on standard of care are 2.50 (Table F1 in the Appendix of the supplemental material). Meanwhile, the expected QALYs in the general population, calculated based on age and gender matching with the patient population in the PRECIOUS trial, are 6.61.^23^ This equates to an absolute QALY loss of 4.11 and consequently a proportional shortfall of 0.62 for the current patient population. This proportional shortfall lies within the 0.41 to 0.70 range, which corresponds to a willingness-to-pay threshold of 50,000 euros per QALY gained according to the Dutch National Healthcare Institute.^22^

### Uncertainty

**Figure 3:**
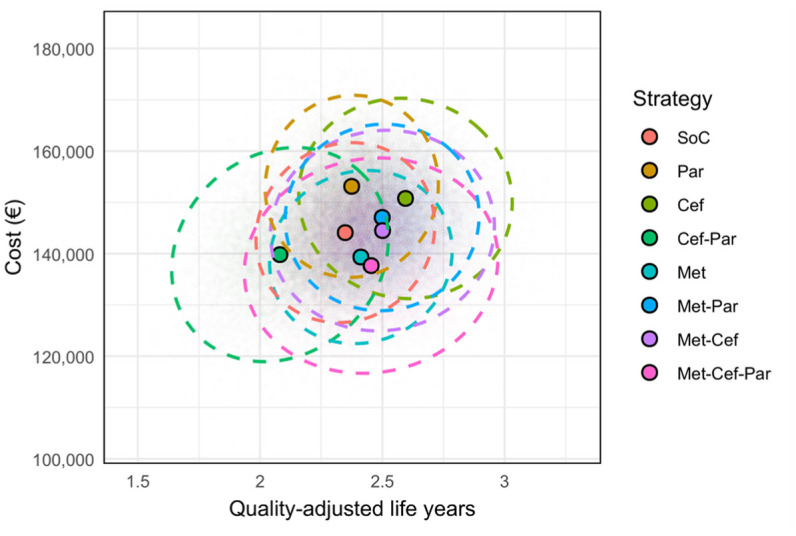
Cost-effectiveness scatterplot. Cef, ceftriaxone; Met, metoclopramide; Par, paracetamol; SoC, standard of care.

The cost-effectiveness scatter plot in Figure 3 illustrates the simulated costs and QALYs for each strategy, providing a visual representation of the uncertainty and overlap between different treatment options.

**Figure 4:**
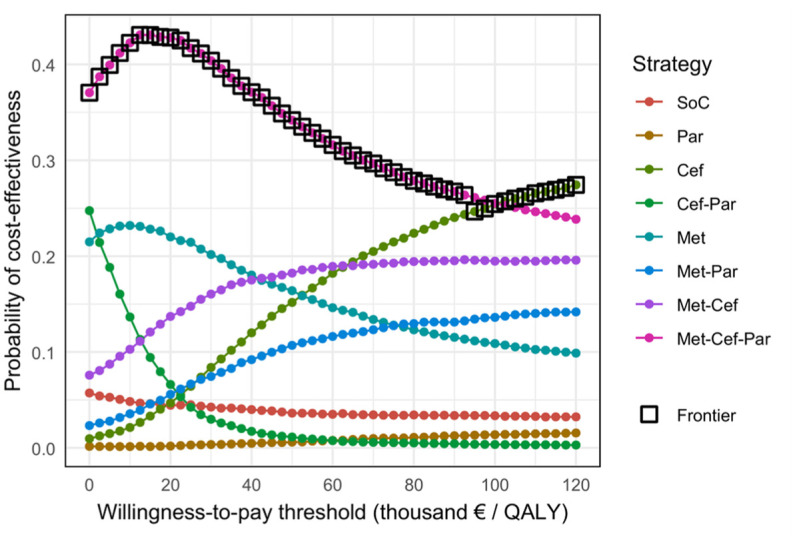
Cost-effectiveness acceptability curve (CEAC) and cost-effectiveness acceptability frontier (CEAF). Cef, ceftriaxone; Met, metoclopramide; Par, paracetamol; SoC, standard of care.

Figure 4 displays the CEACs and CEAF, illustrating both the probability of each treatment strategy being the most cost-effective and the optimal treatment strategy, respectively, across various willingness-to-pay thresholds. At thresholds of 20,000, 50,000, and 80,000 euros per QALY gained, combination treatment with metoclopramide, ceftriaxone, and paracetamol is the optimal treatment, with a 43%, 34% an,d 28% probability of being the most cost-effective treatment option, respectively.

### Value of Information

**Figure 5:**
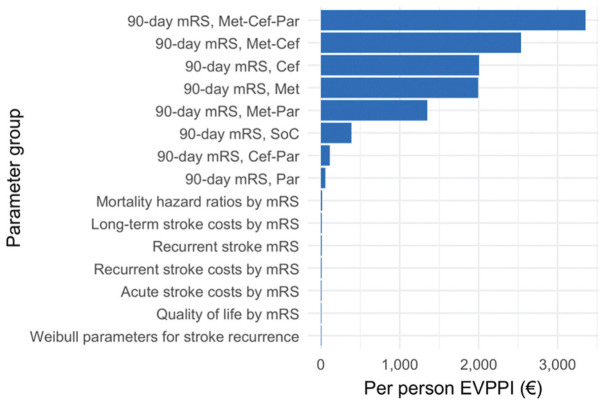
Per-person expected value of partial perfect information (EVPPI) measured in net monetary benefit (euros) for groups of input parameters given a willingness-to-pay threshold of 50,000 euros per QALY gained. Cef, ceftriaxone; Met, metoclopramide; mRS, modified Rankin Scale; Par, paracetamol; SoC, standard of care.

The per-person EVPPI for groups of model parameters, given a willingness-to-pay threshold of 50,000 euros per QALY gained, is presented in Figure 5. The per-person EVPPI for all model input parameters jointly, which is equivalent to the EVPI, is 8,959 euros. The 8 greatest EVPPI values correspond to the sets of acute phase mRS probabilities for the 8 different treatment options. The greatest EVPPI is observed for the set of acute phase mRS probabilities for combination treatment with metoclopramide, ceftriaxone, and paracetamol at 3,353 euros, followed by the mRS probabilities for combination treatment with metoclopramide and ceftriaxone at 2,537 euros, and ceftriaxone monotherapy, with an EVPPI of 2,004 euros.

**Figure 6:**
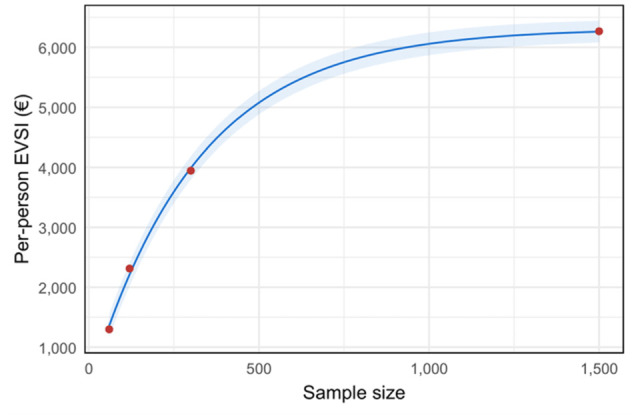
Per-person expected value of sample information (EVSI) measured in net monetary benefit (euros) for a hypothetical new study collecting additional data on acute phase modified Rankin Scale scores for the 8 treatment strategies considered in the PRECIOUS trial.

Figure 6 illustrates the per-person EVSI for a hypothetical new study collecting additional data on acute phase mRS scores for the 8 treatment strategies considered in the PRECIOUS trial. As expected, the EVSI demonstrates diminishing marginal returns for increasing sample sizes, ranging from 1,697 euros to 7,549 euros, corresponding to sample sizes from 160 to 4,000 patients.

**Figure 7:**
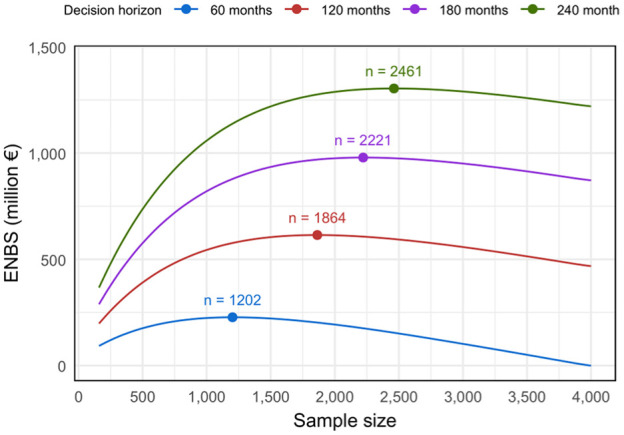
Expected net benefit of sampling (ENBS) measured in net monetary benefit (euros) for a hypothetical new study collecting additional data on acute phase modified Rankin Scale scores for the 8 treatment strategies considered in the PRECIOUS trial.

Figure 7 presents the ENBS for the hypothetical new study across decision horizons from 60 to 240 mo. Initially, the ENBS increases with larger sample sizes as the EVSI increases. However, the ENBS eventually declines with larger sample sizes, as the population that can benefit from the additional research declines with longer trial durations and the overall costs of the trial increase. The positive ENBS values indicate a substantial net value in conducting a new study that collects additional data on acute phase mRS scores. The optimal sample sizes that maximize the ENBS range from 1,202 to 2,461 for decision horizons spanning from 60 to 240 mo, corresponding to ENBS values ranging from 227 to 1,304 million euros.

As a sensitivity analysis, we also evaluated a more focused trial design including only the 3 strategies with the highest EVPPI values (see Appendix H). This analysis indicated that a more targeted trial would have substantially smaller optimal sample sizes, ranging from 779 for a 60-mo decision horizon to 1,184 for a 240-mo horizon. The overall ENBS was similar, with the focused trial yielding a somewhat higher ENBS for shorter decision horizons (60 and 120 mo) and a somewhat lower ENBS for longer horizons (180 and 240 mo).

## Discussion

In this cost-effectiveness analysis, we integrated long-term observational data with data from the PRECIOUS randomized controlled trial.^3^ Our findings suggest that prophylactic treatment with metoclopramide, paracetamol, and ceftriaxone may be the most cost-effective option for preventing infections and fever in older patients with acute stroke from a Dutch health care payer perspective, as it is expected to result in lower costs and higher QALYs compared with standard care. However, there remains substantial uncertainty about the most cost-effective treatment option.

Ceftriaxone monotherapy achieved the best survival outcomes and the highest overall QALYs gained among the treatment strategies. However, combination therapy with metoclopramide, paracetamol, and ceftriaxone resulted in the lowest costs primarily because it achieved the greatest proportion of patients in mRS 0–1 health states, which are associated with no symptoms or significant disability and require minimal stroke care. Although these medications have minor direct costs, their use influences long-term care costs and QALYs by altering the distribution of patients across mRS states in the acute phase. Even modest changes in the distribution across mRS states can have relevant long-term economic and health implications, highlighting the importance of economic evaluations in understanding the broader impact of interventions.^7,49^

The results of our study are in line with another economic evaluation of prophylactic use of ceftriaxone in Dutch stroke patients with a time horizon of 90 d.^50^ Like our study, they found that ceftriaxone improved QALYs compared with standard of care. However, unlike our findings, they found ceftriaxone to also be cost-saving compared with standard of care. Two recent studies by Nguyen et al.^11,12^ and by van Voorst et al.^34^ have conducted model-based cost-effectiveness analyses of different stroke treatments, adopting the same Dutch health care payer perspective as we did in our study. Reported total expected costs for standard of care in these studies ranged roughly from 80,000 to 125,000 euros and expected QALYs from 2.42 to 3.96. Nevertheless, drawing direct comparisons is challenging due to differences in the other studies’ patient populations (which were younger), interventions, time horizons (which were shorter), and analysis settings.

EVPPI analyses for relevant groups of model parameters in our study revealed that decision uncertainty was largely driven by uncertainty about the acute phase mRS probabilities for the treatment options, which represent the relative treatment effects. However, this did not apply to paracetamol used as monotherapy and in combination with ceftriaxone, as these treatment options were unlikely to be cost-effective. As such, uncertainty about the acute phase mRS probabilities for paracetamol used as monotherapy and in combination with ceftriaxone is unlikely to change the optimal decision. Uncertainty about acute and long-term care costs, health-related quality of life, the risk of stroke recurrence, and mortality hazard ratios had a minimal impact on decision uncertainty. This can partly be attributed to the model structure, which, while allowing for treatment-specific acute phase mRS probabilities as observed in the PRECIOUS trial, assumes that patients in similar health states, according to mRS, have a similar prognosis in terms of long-term costs, mortality, and health-related quality of life.

The ENBS analysis suggested that further research to reduce decision uncertainty is highly worthwhile. For a new trial replicating the 8-arm design of PRECIOUS, our analysis indicates the optimal sample size would range from 1,202 to 2,461, depending on the decision horizon. The high ENBS values result from the substantial expected value of reducing uncertainty combined with the high incidence of stroke, which makes the trial costs appear small by comparison. Although we did not aim to determine the optimal trial design across all possible configurations, sensitivity analysis suggests that a focused trial targeting only strategies with the highest EVPPI values could be a valuable pragmatic alternative. While the net value may be similar to that of a full 8-arm trial, the focused design would be easier and less costly to implement, as it may require only half the sample size.

Our study has some limitations. First, we derived long-term care costs from the MR CLEAN trial,^34^ which had a follow-up period of 2 y and recruited a younger and more severe cohort of patients than PRECIOUS did. Consequently, if the ongoing long-term care costs for each mRS health state had not stabilized during this period, our study would not have captured potential changes beyond this time frame. Second, the long-term mortality hazard ratios reported by Huybrechts et al.^28^ were estimated from data collected between 1992 and 2004. Although advancements in stroke care might have reduced acute phase mortality in recent decades, it remains uncertain whether long-term mortality beyond the acute phase has changed. A systematic review and meta-analysis of 122 longitudinal cohort studies by Poon et al.^51^ concluded that long-term survival rates did not appear to have changed over time. Third, we conducted our analysis from a Dutch health care perspective. While a societal perspective could capture the broader societal effects of stroke, such as productivity losses, these are largely irrelevant for our study’s patient population, which had a mean age of 80 y. Lastly, we estimated long-term stroke recurrence risks for patients with ischemic stroke without differentiation for intracerebral hemorrhage. This approach was based on findings from a Danish nationwide registry study,^24^ which reported similar overall risks of stroke recurrence for intracerebral hemorrhage and ischemic stroke. Furthermore, the patient population in the PRECIOUS trial was composed primarily of ischemic stroke cases, accounting for 86% of all cases.

## Conclusion

Our economic evaluation suggests that combination therapy with metoclopramide, paracetamol, and ceftriaxone may be the most cost-effective option for treating older patients with stroke in the Netherlands. However, there is likely great value in reducing uncertainty by conducting a new study that collects additional data on acute phase mRS scores.

## Supplemental Material

sj-pdf-1-mpp-10.1177_23814683251386472 – Supplemental material for Cost-Effectiveness of Metoclopramide, Paracetamol, and Ceftriaxone for the Prevention of Infections and Fever in Elderly Patients with Acute Stroke Supplemental material, sj-pdf-1-mpp-10.1177_23814683251386472 for Cost-Effectiveness of Metoclopramide, Paracetamol, and Ceftriaxone for the Prevention of Infections and Fever in Elderly Patients with Acute Stroke by Mathyn Vervaart, Jeroen C. de Jonge, Philip M. Bath, Hans Olav Melberg, Hendrik Reinink, Wouter M. Sluis, Lisa J. Woodhouse, H. Bart van der Worp and Anne Hege Aamodt in MDM Policy & Practice
