# Supplementary material for: Cost-Effectiveness of Metoclopramide, Paracetamol, and Ceftriaxone for the Prevention of Infections and Fever in Elderly Patients with Acute Stroke
Source: MDM Policy Pract. 2026 Jan 17;11(1):23814683251386472. doi: 10.1177/23814683251386472 (PMC12812187; doi:10.1177/23814683251386472)
Supplement: sj-pdf-1-mpp-10.1177_23814683251386472 – Supplemental material for Cost-Effectiveness of Metoclopramide, Paracetamol, and Ceftriaxone for the Prevention of Infections and Fever in Elderly Patients with Acute Stroke [file sj-pdf-1-mpp-10.1177_23814683251386472.pdf]

Cost-effectiveness of Metoclopramide, Paracetamol and Ceftriaxone for the Prevention of Infections and Fever in Older Patients with Acute Stroke. Supplementary online material.

24 September, 2025

Appendices

Appendix A - CHEERS-VOI checklist[1]

Title and Abstract

- **Item 1: Title:**
  - Cost-effectiveness of Metoclopramide, Paracetamol and Ceftriaxone for the Prevention of Infections and Fever in Older Patients with Acute Stroke.
- **Item 2: Abstract:**
  - A structured abstract is provided at the beginning of the manuscript.

Introduction

- **Item 3: Background and objectives:**
  - The background and objectives are detailed in the Introduction section of the manuscript.

Methods

- **Item 4: Health economic analysis plan:**
  - A pre-specified health economic analysis plan was not developed for this study. The analysis was pre-planned as part of the PRECIOUS trial[2].
- **Item 5: Study population:**
  - The study population consists of patients aged 66 years or older with acute ischemic stroke or intracerebral hemorrhage, and a National Institutes of Health Stroke Scale (NIHSS) score of ≥ 6.
  - The population for the population-level EVSI was based on a monthly incidence of 1,291 eligible patients. This was derived from the total annual stroke incidence in the Netherlands in 2023 (41,300 patients)[3] and adjusted to reflect the proportion of patients meeting the trial's eligibility criteria: approximately 75% are aged ≥ 66 years[4,5] and 50% have a NIHSS ≥ 6[6]. The ENBS analysis considered decision horizons of 60, 120, 180, and 240 months.
- **Item 6: Setting and location:**
  - The analysis was conducted from a Dutch healthcare perspective, as most patients in the PRECIOUS trial were from the Netherlands.
- **Item 7: Comparators:**

- The eight treatment strategies from the randomised $2 \times 2 \times 2$ factorial PRECIOUS trial were compared[2]: metoclopramide, ceftriaxone, and paracetamol as monotherapies or in all possible combinations, versus standard care, compared to standard care only.

- **Item 8: Perspective:**
  - We adopted a Dutch healthcare payer perspective. A societal perspective was not chosen as productivity losses are largely irrelevant for this patient population with a mean age of 80 years.

- **Item 9: Time horizon:**
  - We used a time horizon of 20 years, until a maximum age of 100 years or death, whichever occurred first.

- **Item 10: Discount rate:**
  - Costs and QALYs were discounted at 3% and 1.5% per year, respectively, following the Dutch guidelines for economic evaluations in healthcare[7]. Population-level ENBS estimates were discounted at an annual rate of 3%.

- **Item 11: Selection of outcomes:**
  - Quality-Adjusted Life Years (QALYs) were the primary measure of health outcome.

- **Item 12: Measurement of outcomes:**
  - Health-related quality of life was measured using the EQ-5D-5L instrument[8] at 90 days of follow-up in the PRECIOUS trial. Life years were estimated using a state-transition model informed by trial and literature data.

- **Item 13: Valuation of outcomes:**
  - Utility weights were derived by applying Dutch tariffs[9] to the EQ-5D-5L data to value quality of life. QALYs were then computed by combining utility weights with time spent in health states[10].

- **Item 14: Measurement and valuation of resources and costs:**
  - Costs were valued from a Dutch healthcare payer perspective. Resource use for the acute phase was primarily informed by patient-level data from the PRECIOUS trial and supplemented with published literature, while long-term care costs were sourced from external studies. Resource use was valued using unit costs from sources including the Dutch costing manual, Dutch list prices and other Dutch studies. The analysis included costs for drugs, the acute phase, long-term care, and age-related increases in indirect medical costs, with full details provided in the Methods section.

- **Item 15: Currency, price date, and conversion:**

– All costs were updated to 2024 euros using the medical component of the Dutch consumer price index.

- **Item 16: Rationale and description of model:**
  – We developed a decision model consisting of a decision tree for the initial 90 days post-stroke and a state-transition model for long-term consequences. This two-component structure with mRS-based health states allows for the synthesis of short-term trial outcomes with long-term prognosis data from the literature and is common in stroke economic evaluations[11–14]. The model is not publicly available.

- **Item S1: VOI estimation methods:**
  – The EVPI was estimated from the 10,000 probabilistic analysis simulations by averaging the maximum Net Monetary Benefit (NMB) across strategies. The EVPPI and EVSI were calculated using a nonparametric regression approach with Bayesian Additive Regression Trees (BART) as implemented in the `voi` R package[15]. The standard error of these estimates were derived from the posterior standard deviation of the BART model. EVSI results were calculated for four sample sizes and interpolated using an asymptotic regression model, standard errors were interpolated using smoothing splines[16].

- **Item 17: Analytics and assumptions:**
  – A cost-effectiveness threshold of €50,000 per QALY gained was used for the main VOI analysis. This was based on Dutch guidelines[17], where the willingness-to-pay threshold is linked to disease severity as quantified by proportional shortfall (0.62 in this patient population).

- **Item S2: Evidence base:**
  – The evidence base for the analysis consists of patient-level data from the PRECIOUS randomized controlled trial[2] to inform model input parameters related to treatment effects, quality of life weights, and acute phase resource use. This trial data was supplemented with data from published literature and registries for long-term parameters (e.g., mortality, stroke recurrence, costs). Potential for heterogeneity exists between the trial population and the populations from which these long-term data were derived. These limitations are further detailed in the main manuscript's Discussion section.

- **Item 18: Characterizing heterogeneity:**
  – No subgroup analyses were conducted for this economic evaluation.

- **Item 19: Characterizing distributional effects:**
  – Distributional effects were not considered in this analysis.

- **Item 20: Characterizing uncertainty:**

  - Parameter uncertainty was characterized using probability distributions derived from the PRECIOUS trial data and the literature, as detailed in Appendix B. A full probabilistic analysis with 10,000 Monte Carlo simulations was conducted.

- **Item S3: Parameters of interest in VOI analysis:**
  - EVPPI was computed for multiple parameter groups to identify key drivers of uncertainty. These groups included: the eight sets of 90-day mRS distribution parameters (representing treatment effects); long-term prognosis parameters (mortality hazard ratios, stroke recurrence rates); mRS-specific costs (acute, long-term, and for recurrent stroke); and quality of life weights. The EVSI and ENBS were subsequently computed for collecting new data on the 90-day mRS distributions only, as the EVPPI analysis identified these as the primary source of decision uncertainty.

- **Item S4: Study design(s) proposed in VOI analysis:**
  - The VOI analysis was an exploratory assessment and did not aim to formally determine an optimal trial design. We evaluated two hypothetical Randomized Controlled Trial (RCT) designs with even allocation across arms: (1) an eight-arm trial mimicking the PRECIOUS trial, and (2) as a sensitivity analysis, a more focused three-arm trial including only the strategies with the highest EVPPI values. For both designs, the proposed research would collect additional data on the 90-day mRS distributions, with sample sizes of up to 500 patients per arm considered. We assumed an enrollment rate of 80 patients per month, a follow-up period of 3 months, and an analysis and reporting period of 6 months, based on the PRECIOUS trial protocol. We adjusted the decision horizons for the recruitment period, follow-up, and analysis and reporting period.

- **Item S5: Data generation:**
  - Data for the EVSI calculation were simulated to represent potential outcomes of a future trial. For each simulation, a set of probabilities for the seven mRS outcomes was first drawn from the Dirichlet distributions for the mRS parameters. This set of probabilities was then used to generate the number of patients in each mRS category for each trial arm by drawing from a multinomial distribution. The simulation assumed even allocation of patients to each treatment strategy.

- **Item S6: Costs of research studies for ENBS:**
  - The costs for the hypothetical research study were based on the costs of the PRECIOUS trial, including fixed costs of €4.6 million and variable per-patient costs of €500.

- **Item 21: Approach to engagement with patients and others affected by the study:**
  - Clinicians and researchers from the PRECIOUS trial provided expert input on the design and key assumptions of this economic evaluation. Patients or the public were not engaged in this process.

Results

- **Item 22: Study parameters:**
  - All model input parameters, their values, distributions, and data sources are reported in Table B1 in the Appendix.
- **Item 23: Summary of main results:**
  - The mean costs, QALYs, and NMB for each strategy are reported in the Results section (Table 2) of the main manuscript. The results for the VOI measures (EVPI, EVPPI, EVSI, and ENBS) are also presented in the Results section, under the 'Value of information' subsection and in Figures 5, 6, and 7.
- **Item 24: Effect of uncertainty:**
  - The effect of parameter uncertainty is presented in the cost-effectiveness scatterplot (Figure 3) and the cost-effectiveness acceptability curves/frontier (Figure 4). A sensitivity analysis assessed the impact of using a common discount rate for costs and effects.
- **Item 25: Effect of engagement with patients and others affected by the study:**
  - Input from engaged clinical experts informed the clinical plausibility of the economic model, including its structure, key assumptions, and alignment with the stroke care pathway.

Discussion

- **Item 26: Study findings, limitations, generalizability, and current knowledge:**
  - The main findings, limitations, and generalizability of the study are presented in the Discussion section of the main manuscript.

Other

- **Item 27: Source of funding:**
  - This study was funded by the European Union's Horizon 2020 research and innovation programme (634809).
- **Item 28: Conflicts of interest:**
  - MV, HOM, LJW: none related. JCdJ and WMS report grants from the European Union, all paid to their institution. PMB reports having received grants from the UK National Institute of Health Research, and fees as consultant from CoMind, DiaMedica, Phagenesis and Roche. AHA reports research grants from Boehringer Ingelheim, lectures fee from

Abbvie, BMS/Pfizer, Novartis, Roche and Teva and participation in Advisory Board for Lundbeck, Abbvie and MSD; none related. HBvdW reports having received grants from the European Union, the Dutch Heart Foundation, and Stryker for research, and funding for consultancy from Bayer and TargED, all paid to his institution.

Appendix B - Model input parameters and distributions

Table B1: Model parameter distributions and data sources

Parameter description	Mean, $\boldsymbol{\mu}$	Covariance, $\boldsymbol{\Sigma}$	Distribution	Source
**90-day mRS**				
By treatment arm	Table D1		Dirichlet	PRECIOUS trial[2]
**Stroke recurrence rate**				
Weibull log shape	-0.57	$0.03 \quad -0.00$	Bivariate	Skajaa et al.[18]
Weibull log scale	5.49	$-0.00 \quad 0.11$	Normal	
Recurrence HR mRS 1-2 vs. 0	1.18	0.09	Lognormal	De Havenon et al.[19]
Recurrence HR mRS 3+ vs. 0	1.36	0.12	Lognormal	De Havenon et al.[19]
**General population mortality rate**				
For ages 80 - 99	$0.001 - 0.008$		Constant	Statistics Netherlands[20]
**Mortality hazard ratios by mRS**				
mRS 0 vs. general population	1.200	0.085	Lognormal	Shavelle et al.[21]
mRS 1 vs. 0	1.175	0.085	Lognormal	Huybrechts et al.[22]
mRS 2 vs. 1	1.320	0.085	Lognormal	Huybrechts et al.[22]
mRS 3 vs. 2	1.162	0.087	Lognormal	Huybrechts et al.[22]
mRS 4 vs. 3	1.426	0.295	Lognormal	Huybrechts et al.[22]
mRS 5 vs. 4	2.226	1.109	Lognormal	Huybrechts et al.[22]
**Utility weights**				
mRS 0	0.939	0.013	Beta	PRECIOUS trial[2]
mRS 1	0.847	0.012	Beta	PRECIOUS trial[2]
mRS 2	0.780	0.012	Beta	PRECIOUS trial[2]
mRS 3	0.594	0.017	Beta	PRECIOUS trial[2]
mRS 4	0.300	0.022	Beta	PRECIOUS trial[2]
mRS 5	0.144	0.020	Beta	PRECIOUS trial[2]
mRS 6	0	0	Constant	PRECIOUS trial[2]
**Drug costs (€)**				
Metoclopramide, 3 x 10 mg	0.27		Constant	Dutch list prices[23]
Paracetamol, 3 x 1,000 mg	0.30		Constant	Dutch list prices[23]
Ceftriaxone, 1,000 mg	9.34		Constant	Dutch list prices[23]
**Drug dosing and duration**				
Metoclopramide, daily dose (mg)	30		Constant	PRECIOUS trial[2]
Metoclopramide, duration (days)	4		Constant	PRECIOUS trial[2]
Paracetamol, daily dose (mg)	4,000		Constant	PRECIOUS trial[2]
Paracetamol, duration (days)	4		Constant	PRECIOUS trial[2]
Ceftriaxone, daily dose (mg)	2,000		Constant	PRECIOUS trial[2]
Ceftriaxone, duration (days)	4		Constant	PRECIOUS trial[2]
**Acute phase length of stay by mRS (days)**				
Hospital	Table D2		Gamma	PRECIOUS trial[2]
Rehabilitation centre	Table D2		Gamma	PRECIOUS trial[2]
Nursing home	Table D2		Gamma	PRECIOUS trial[2]
Home	Table D2		Gamma	PRECIOUS trial[2]
**Acute phase procedures (proportion)**				
Intravenous thrombolysis by mRS	Table D2		Beta	PRECIOUS trial[2]
Mechanical thrombectomy by mRS	Table D2		Beta	PRECIOUS trial[2]
Carotid endarterectomy	0.065	0.013	Beta	Buisman et al.[24]
**Acute phase unit costs (€)**				

Continued on next page

Hospital, per day	683	137	Gamma	Dutch costing manual[25]
Rehabilitation centre, per day	379	76	Gamma	Dutch costing manual[25]
Nursing home, per day	308	62	Gamma	Dutch costing manual[25]
Visits, per patient	250	50	Gamma	Buisman et al.[24]
Diagnostics and imaging, per patient	586	117	Gamma	Buisman et al.[24]
Allied health services, per patient	257	51	Gamma	Buisman et al.[24]
Carotid endarterectomy	5,426	1,085	Gamma	Buisman et al.[24]
Intravenous thrombolysis	1,130	226	Gamma	van den Berg et al.[26]
Endovascular thrombectomy	11,797	2,359	Gamma	van den Berg et al.[26]
Intravenous infusion	180	36	Gamma	NICE TA650[27]
**Long-term care costs, per week (€)**				
mRS 0	127	21	Gamma	Voorst et al.[28]
mRS 1	127	21	Gamma	Voorst et al.[28]
mRS 2	183	20	Gamma	Voorst et al.[28]
mRS 3	412	22	Gamma	Voorst et al.[28]
mRS 4	925	62	Gamma	Voorst et al.[28]
mRS 5	1,208	33	Gamma	Voorst et al.[28]
**Indirect medical costs, per week (€)**				
Age-related changes, age 80 - 99	0 – 704		Constant	PAID 3.0[29]
**Patient characteristics**				
Mean age at start of model	80		Constant	PRECIOUS trial[2]
Proportion men	0.50		Constant	PRECIOUS trial[2]
**Other parameters**				
Annual discount rate for costs	0.030		Constant	Dutch guidelines[7]
Annual discount rate for effects	0.015		Constant	Dutch guidelines[7]
Time horizon in years	20		Constant	Lifetime
Model cycles per year	52		Constant	Weekly

mRS, modified Rankin Scale. All costs were converted to 2024 euros using the medical care component of the consumer price index.

Appendix C - Modelling of Key Clinical Events

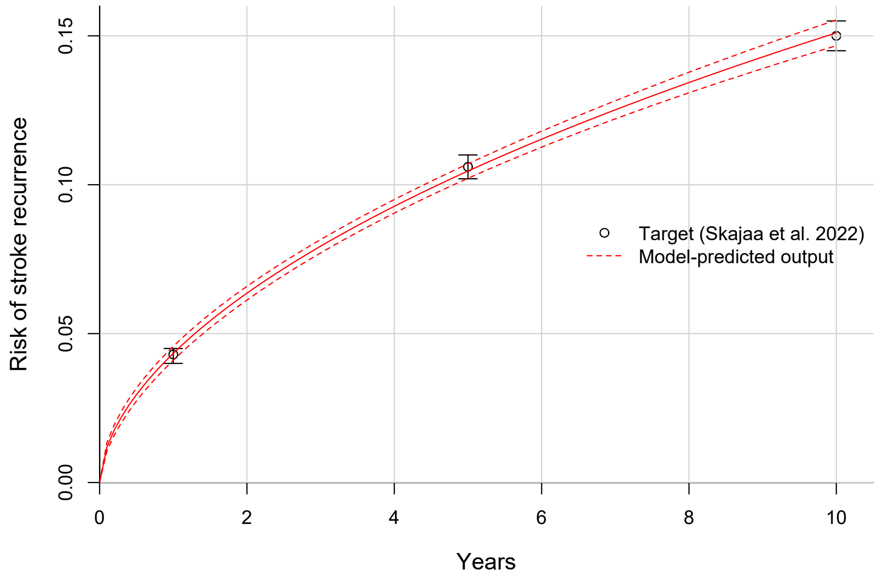

Figure C1: Results of the Bayesian calibration of a Weibull model for estimating stroke recurrence risks at 1, 5, and 10-year intervals, based on data from Skajaa et al. (2022).

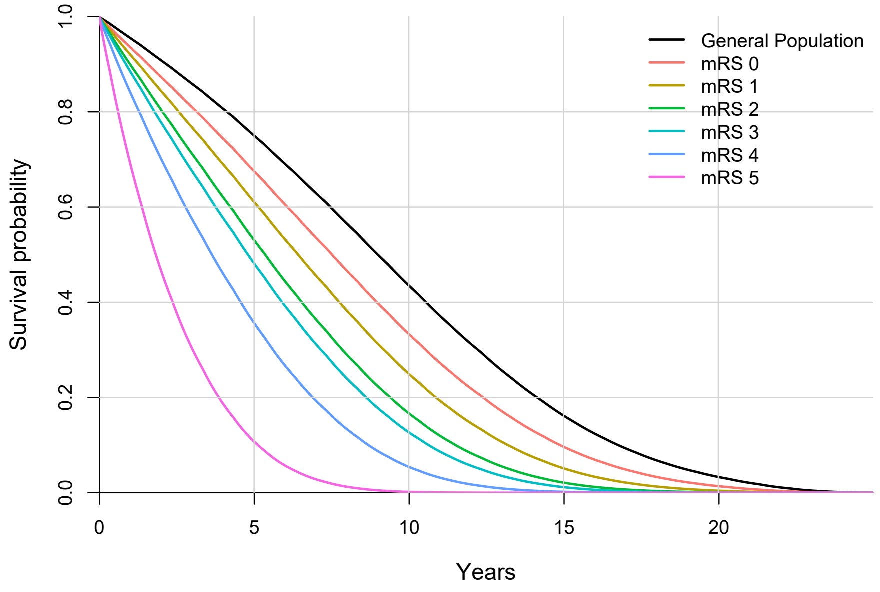

Figure C2: Survival by modified Rankin scale at 90 days post-stroke

Appendix D - Complete Case Values and Missing Data Summary

Table D1: Distribution of modified rankin scale by treatment arm at 90 days of follow-up in PRECIOUS (complete case values)

**Characteristic**	**Overall**	**SoC**	**Par**	**Cef**	**Cef-Par**	**Met**	**Met-Par**	**Met-Cef**	**Met-Cef-Par**
Missing, No. (%)	21 (1.4%)	5 (2.0%)	1 (0.5%)	3 (1.8%)	1 (0.7%)	5 (2.2%)	3 (1.5%)	3 (2.1%)	0 (0.0%)
Complete cases, No. (%)	1,469 (98.6%)	241 (98.0%)	219 (99.5%)	166 (98.2%)	151 (99.3%)	222 (97.8%)	202 (98.5%)	139 (97.9%)	129 (100.0%)
mRS, No. (%)									
0. No symptoms	70 (4.8%)	17 (7.1%)	5 (2.3%)	10 (6.0%)	7 (4.6%)	8 (3.6%)	10 (5.0%)	4 (2.9%)	9 (7.0%)
1. No significant disability	139 (9.5%)	17 (7.1%)	23 (10.5%)	15 (9.0%)	10 (6.6%)	23 (10.4%)	22 (10.9%)	16 (11.5%)	13 (10.1%)
2. Slight disability	180 (12.3%)	22 (9.1%)	22 (10.0%)	28 (16.9%)	14 (9.3%)	30 (13.5%)	26 (12.9%)	20 (14.4%)	18 (14.0%)
3. Moderate disability	286 (19.5%)	51 (21.2%)	54 (24.7%)	25 (15.1%)	28 (18.5%)	45 (20.3%)	37 (18.3%)	29 (20.9%)	17 (13.2%)
4. Moderately severe disability	263 (17.9%)	46 (19.1%)	42 (19.2%)	37 (22.3%)	29 (19.2%)	29 (13.1%)	38 (18.8%)	20 (14.4%)	22 (17.1%)
5. Severe disability	206 (14.0%)	30 (12.4%)	32 (14.6%)	20 (12.0%)	20 (13.2%)	36 (16.2%)	28 (13.9%)	22 (15.8%)	18 (14.0%)
6. Dead	325 (22.1%)	58 (24.1%)	41 (18.7%)	31 (18.7%)	43 (28.5%)	51 (23.0%)	41 (20.3%)	28 (20.1%)	32 (24.8%)
mRS 0-2 vs. 3-6, No. (%)									
mRS 0-2	389 (26.5%)	56 (23.2%)	50 (22.8%)	53 (31.9%)	31 (20.5%)	61 (27.5%)	58 (28.7%)	40 (28.8%)	40 (31.0%)
mRS 3-6	1,080 (73.5%)	185 (76.8%)	169 (77.2%)	113 (68.1%)	120 (79.5%)	161 (72.5%)	144 (71.3%)	99 (71.2%)	89 (69.0%)
mRS, Median (Q1, Q3)	4 (2, 5)	4 (3, 5)	4 (3, 5)	4 (2, 5)	4 (3, 6)	4 (2, 5)	4 (2, 5)	4 (2, 5)	4 (2, 5)

Cef, Ceftriaxone; Par, Paracetamol; Met, Metoclopramide; mRS, Modified Rankin Scale; SoC, Standard of Care.

Table D2: Summary of EQ-5D-5L values, intravenous thrombolysis, mechanical thrombectomy, and length of stay by modified rankin scale at 90 days of follow-up in PRECIOUS (complete case values)

**Characteristic**	**Overall**<br>N = 1,469	**mRS 0**<br>N = 70	**mRS 1**<br>N = 139	**mRS 2**<br>N = 180	**mRS 3**<br>N = 286	**mRS 4**<br>N = 263	**mRS 5**<br>N = 206	**mRS 6**<br>N = 325
EQ-5D-5L								
Mean (SE)	0.42 (0.01)	0.94 (0.01)	0.85 (0.01)	0.78 (0.01)	0.59 (0.02)	0.30 (0.02)	0.15 (0.03)	0.00 (0.00)
N Missing (% Missing%)	375 (25.5%)	6 (8.6%)	22 (15.8%)	22 (12.2%)	74 (25.9%)	113 (43.0%)	138 (67.0%)	0 (0.0%)
Intravenous thrombolysis, No. (%)								
Yes	592 (40.3%)	28 (40.0%)	68 (48.9%)	93 (51.7%)	125 (43.7%)	90 (34.2%)	69 (33.5%)	119 (36.6%)
No	679 (46.2%)	38 (54.3%)	59 (42.4%)	71 (39.4%)	123 (43.0%)	119 (45.2%)	105 (51.0%)	164 (50.5%)
Missing	198 (13.5%)	4 (5.7%)	12 (8.6%)	16 (8.9%)	38 (13.3%)	54 (20.5%)	32 (15.5%)	42 (12.9%)
Mechanical thrombectomy, No. (%)								
Yes	304 (20.7%)	12 (17.1%)	37 (26.6%)	46 (25.6%)	52 (18.2%)	55 (20.9%)	44 (21.4%)	58 (17.8%)
No	967 (65.8%)	54 (77.1%)	90 (64.7%)	118 (65.6%)	196 (68.5%)	154 (58.6%)	130 (63.1%)	225 (69.2%)
Missing	198 (13.5%)	4 (5.7%)	12 (8.6%)	16 (8.9%)	38 (13.3%)	54 (20.5%)	32 (15.5%)	42 (12.9%)
Length of stay (days), Mean (SE)								
Hospital	17.1 (0.50)	8.3 (1.39)	9.0 (0.72)	13.1 (1.04)	17.3 (0.99)	22.5 (1.49)	27.8 (1.80)	13.3 (0.72)
Rehabilitation centre	18.5 (0.72)	4.2 (1.56)	6.5 (1.08)	13.3 (1.65)	25.8 (1.68)	34.5 (1.99)	29.0 (2.33)	3.4 (0.63)
Nursing home	4.4 (0.39)	0.0 (0.00)	0.5 (0.55)	0.4 (0.31)	1.9 (0.59)	7.2 (1.21)	14.4 (1.78)	2.7 (0.52)
Home	24.9 (0.88)	64.3 (3.79)	56.3 (2.94)	47.5 (2.57)	31.0 (1.90)	13.2 (1.55)	11.7 (1.72)	2.9 (0.64)
Missing	10.9 (0.62)	13.2 (2.96)	17.6 (2.48)	15.8 (2.13)	14.1 (1.54)	12.6 (1.48)	7.1 (1.34)	3.0 (0.74)

mRS, Modified Rankin Scale.

Appendix E - Summary of Multiple Imputed Values

We handled missing data using multivariate imputation by chained equations (MICE)[30]. MICE is a statistical technique that replaces missing data with simulated values, thereby avoiding potential bias in statistical analyses and facilitating intention-to-treat analyses. The simulated values are drawn from a series of iterative regression models for each variable with missing data, each conditioned on all the other variables in the imputation model. We specified logistic regression models for binary variables and polytomous regression models for variables with multiple categories and generated 100 imputed data sets[30]. We pooled parameter estimates and standard errors across the 100 imputed data sets by applying Rubin's rules[31], thereby accounting for imputation uncertainty.

Table E1: Multiple imputed distribution of modified rankin scale by treatment arm at 90 days of follow-up in PRECIOUS

**Characteristic**	**SoC**	**Par**	**Cef**	**Cef-Par**	**Met**	**Met-Par**	**Met-Cef**	**Met-Cef-Par**
Modified Rankin Scale, ESS (%)								
0. No symptoms	17.2 (7.2%)	5 (2.3%)	9.9 (6%)	7 (4.7%)	8.1 (3.7%)	10 (4.9%)	4 (2.8%)	8.9 (7%)
1. No significant disability	16.8 (7%)	22.9 (10.5%)	15.1 (9.1%)	9.9 (6.6%)	22.8 (10.4%)	22 (10.8%)	16.4 (11.7%)	12.9 (10.1%)
2. Slight disability	21.8 (9.1%)	22 (10.1%)	28.3 (17%)	14.1 (9.4%)	29.5 (13.5%)	26.1 (12.9%)	20.1 (14.4%)	17.9 (14%)
3. Moderate disability	50.7 (21.2%)	54.3 (24.8%)	25.1 (15.1%)	27.6 (18.5%)	45 (20.5%)	37.4 (18.4%)	29 (20.8%)	16.9 (13.2%)
4. Moderately severe disability	45.5 (19%)	42 (19.2%)	36.8 (22.2%)	28.6 (19.2%)	28.6 (13%)	38.3 (18.9%)	20.1 (14.4%)	21.8 (17.1%)
5. Severe disability	29.8 (12.4%)	31.9 (14.6%)	19.9 (12%)	19.7 (13.2%)	35.6 (16.2%)	28.2 (13.9%)	22.1 (15.8%)	17.9 (14%)
6. Dead	57.4 (24%)	40.9 (18.7%)	30.8 (18.6%)	42.3 (28.4%)	49.6 (22.7%)	41 (20.2%)	28 (20%)	31.8 (24.8%)

Cef, Ceftriaxone; ESS, Effective Sample Size; Par, Paracetamol; Met, Metoclopramide; SoC, Standard of Care.

Table E2: Summary of multiple imputed EQ-5D-5L values, intravenous thrombolysis, mechanical thrombectomy, and length of stay by modified rankin scale at 90 days of follow-up in PRECIOUS

**Characteristic**	**mRS 0**	**mRS 1**	**mRS 2**	**mRS 3**	**mRS 4**	**mRS 5**	**mRS 6**
EQ-5D-5L							
Mean (SE)	0.94 (0.01)	0.85 (0.01)	0.78 (0.01)	0.59 (0.02)	0.3 (0.02)	0.14 (0.02)	0 (0)
Intravenous thrombolysis							
Mean (SE)	0.43 (0.06)	0.54 (0.05)	0.57 (0.04)	0.52 (0.04)	0.44 (0.05)	0.4 (0.05)	0.43 (0.04)
Mechanical thrombectomy							
Mean (SE)	0.2 (0.05)	0.3 (0.04)	0.3 (0.04)	0.24 (0.04)	0.29 (0.05)	0.28 (0.05)	0.23 (0.04)
Length of stay (days), Mean (SE)							
Hospital	8.92 (0.42)	9.82 (0.31)	14.54 (0.42)	18.87 (0.46)	24.90 (0.63)	29.66 (0.69)	14.86 (0.39)
Rehabilitation centre	5.62 (0.81)	7.93 (0.45)	15.95 (0.61)	29.72 (0.74)	39.53 (0.81)	31.21 (0.87)	4.13 (0.35)
Nursing home	0.04 (0.08)	0.72 (0.18)	0.55 (0.15)	2.45 (0.31)	9.00 (0.53)	15.99 (0.68)	3.42 (0.28)
Home	75.42 (0.90)	71.54 (0.57)	58.95 (0.73)	38.97 (0.77)	16.57 (0.67)	13.15 (0.65)	3.54 (0.34)

mRS, Modified Rankin Scale.

Appendix F - Undiscounted cost-effectiveness results

Table F1: Probabilistic cost-effectiveness results (undiscounted). All values are expressed as Mean (Standard Error). The treatment with the greatest net monetary benefit is displayed in bold.

Strategy	Cost	QALYs	NMB, λ=20,000	NMB, λ=50,000	NMB, λ=80,000
SoC	159,206 (7,723)	2.51 (0.16)	-109,103 (8,006)	-33,948 (10,462)	41,206 (14,185)
Par	168,870 (7,791)	2.53 (0.16)	-118,294 (8,210)	-42,430 (10,657)	33,434 (14,250)
Cef	166,755 (8,587)	2.77 (0.19)	-111,403 (9,391)	-28,376 (12,848)	54,652 (17,556)
Cef-Par	153,819 (9,232)	2.22 (0.19)	-109,463 (9,325)	-42,930 (12,091)	23,603 (16,535)
Met	153,777 (7,405)	2.57 (0.16)	-102,340 (7,740)	-25,185 (10,380)	51,970 (14,277)
Met-Par	162,536 (7,989)	2.67 (0.17)	-109,225 (8,504)	-29,260 (11,425)	50,705 (15,598)
Met-Cef	159,504 (8,599)	2.67 (0.20)	-106,194 (9,155)	-26,228 (12,615)	53,737 (17,523)
Met-Cef-Par	152,135 (9,257)	2.62 (0.23)	**-99,718 (9,831)**	**-21,094 (13,822)**	**57,530 (19,467)**

Cef, Ceftriaxone; Met, Metoclopramide; NMB, Net Monetary Benefit; Par, Paracetamol; QALY, Quality-Adjusted Life Year; SE, Standard Error; SoC, Standard of Care; λ, Willingness-to-pay threshold (incremental cost per QALY gained). Costs and NMB are reported in euros.

Appendix G - Cost-effectiveness results with common discount rate

Table G1: Probabilistic cost-effectiveness results with a common discount rate of 3.5% for both costs and effects. All values are expressed as Mean (Standard Error). The treatment with the greatest net monetary benefit is displayed in bold.

Strategy	Cost	QALYs	NMB, λ=20,000	NMB, λ=50,000	NMB, λ=80,000
SoC	142,061 (7,088)	2.17 (0.14)	-98,723 (7,397)	-33,716 (9,437)	31,290 (12,511)
Par	150,976 (7,135)	2.20 (0.13)	-107,055 (7,576)	-41,174 (9,680)	24,708 (12,724)
Cef	148,697 (7,994)	2.40 (0.16)	-100,736 (8,705)	-28,794 (11,552)	43,147 (15,457)
Cef-Par	137,806 (8,548)	1.92 (0.17)	-99,354 (8,713)	-41,676 (11,032)	16,001 (14,742)
Met	137,342 (6,712)	2.23 (0.14)	-92,776 (7,095)	-25,926 (9,414)	40,925 (12,780)
Met-Par	145,035 (7,258)	2.31 (0.15)	-98,882 (7,842)	-29,652 (10,365)	39,577 (13,879)
Met-Cef	142,488 (7,744)	2.31 (0.17)	-96,312 (8,419)	-27,048 (11,500)	42,216 (15,735)
Met-Cef-Par	135,897 (8,418)	2.26 (0.20)	**-90,607 (9,139)**	**-22,671 (12,661)**	**45,264 (17,505)**

Cef, Ceftriaxone; Met, Metoclopramide; NMB, Net Monetary Benefit; Par, Paracetamol; QALY, Quality-Adjusted Life Year; SE, Standard Error; SoC, Standard of Care; λ, Willingness-to-pay threshold (incremental cost per QALY gained). Costs and NMB are reported in euros.

Appendix H - Value of information for a 3-arm trial

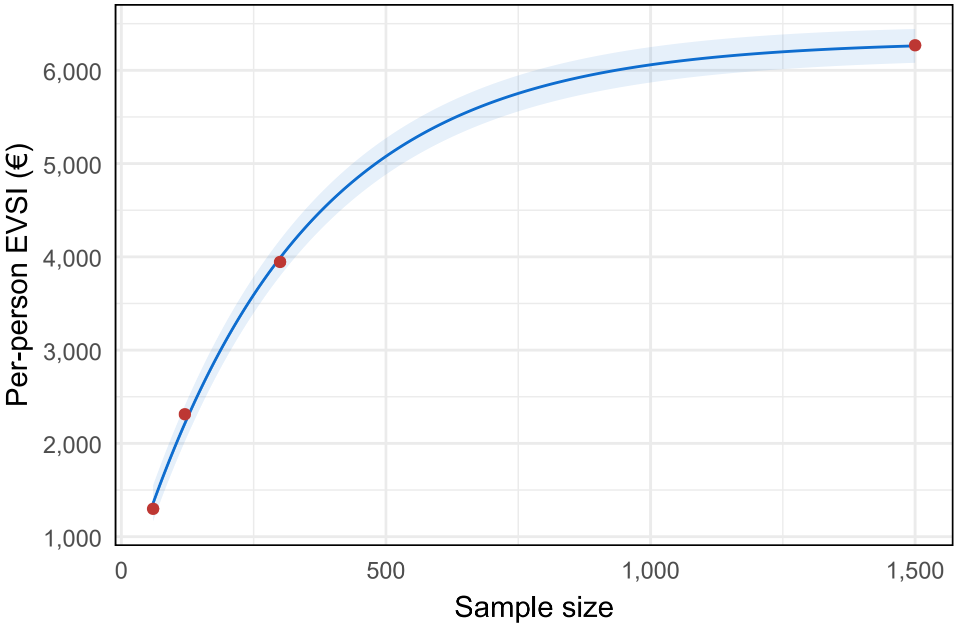

Figure H1: Per person expected value of sample information (EVSI) measured in net monetary benefit (euros) for a hypothetical new study collecting additional data on acute phase mRS scores for the three strategies with the highest EVPPI: ceftriaxone monotherapy, combination therapy with metoclopramide and ceftriaxone, and combination therapy with metoclopramide, ceftriaxone, and paracetamol.

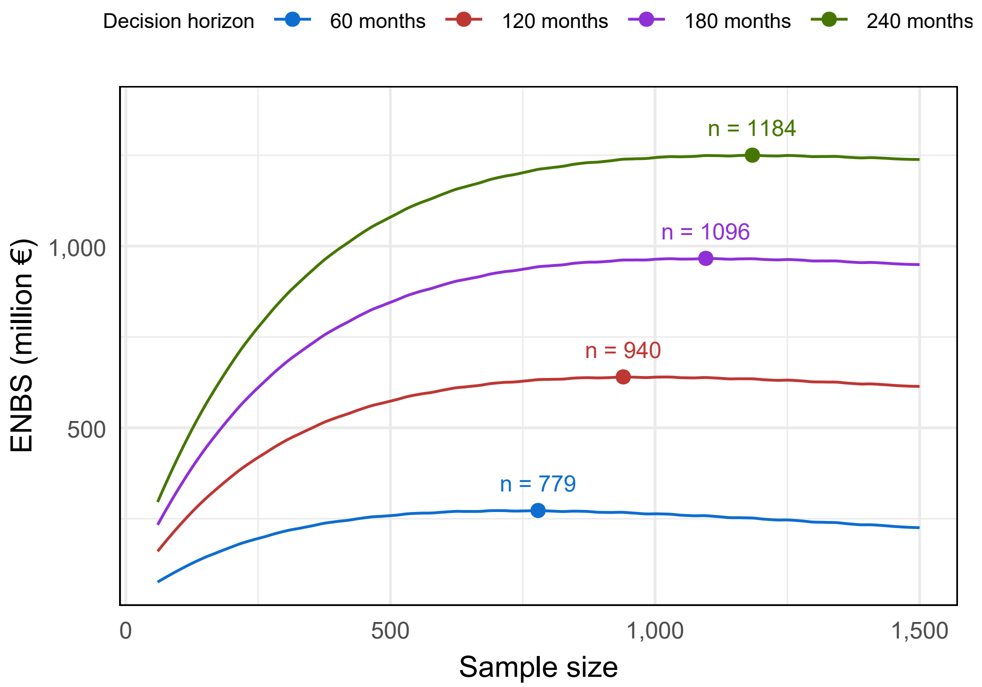

Figure H2: Expected net benefit of sampling (ENBS) measured in net monetary benefit (euros) for a hypothetical new study collecting additional data on acute phase mRS scores for the three strategies with the highest EVPPI: ceftriaxone monotherapy, combination therapy with metoclopramide and ceftriaxone, and combination therapy with metoclopramide, ceftriaxone, and paracetamol.

References

1. Kunst N, Siu A, Drummond M, et al. Consolidated Health Economic Evaluation Reporting Standards - Value of Information (CHEERS-VOI): Explanation and Elaboration. *Value in Health* 2023; 26: 1461–1473.

2. de Jonge JC, Sluis WM, Reinink H, et al. Prevention of infections and fever to improve outcome in older patients with acute stroke (PRECIOUS): A randomised, open, phase III, multifactorial, clinical trial with blinded outcome assessment. *Lancet Reg Health Eur* 2024; 36: 100782.

3. National Institute for Public Health and the Environment (RIVM). Stroke Incidence by Age, Gender, and Type, https://www.vzinfo.nl/beroerte/leeftijd-en-geslacht/huisartsencijfers (2024, accessed 7 March 2025).

4. Heuschmann PU, Wiedmann S, Wellwood I, et al. Three-month stroke outcome: The European Registers of Stroke (EROS) investigators. *Neurology* 2011; 76: 159–165.

5. Feigin VL, Lawes CM, Bennett DA, et al. Stroke epidemiology: A review of population-based studies of incidence, prevalence, and case-fatality in the late 20th century. *The Lancet Neurology* 2003; 2: 43–53.

6. Den Hertog HM, Van Der Worp HB, Van Gemert HMA, et al. The Paracetamol (Acetaminophen) In Stroke (PAIS) trial: A multicentre, randomised, placebo-controlled, phase III trial. *The Lancet Neurology* 2009; 8: 434–440.

7. Dutch National Health Care Institute. *Guideline for economic evaluations in healthcare*, https://english.zorginstituutnederland.nl/about-us/publications/reports/2024/01/16/guideline-for-economic-evaluations-in-healthcare (2024, accessed 5 May 2025).

8. Herdman M, Gudex C, Lloyd A, et al. Development and preliminary testing of the new five-level version of EQ-5D (EQ-5D-5L). *Qual Life Res* 2011; 20: 1727–1736.

9. M Versteegh M, M Vermeulen K, M A A Evers S, et al. Dutch Tariff for the Five-Level Version of EQ-5D. *Value Health* 2016; 19: 343–352.

10. Brazier J. *Measuring and Valuing Health Benefits for Economic Evaluation.* OUP Oxford, 2007.

11. Kunz WG, Hunink MGM, Sommer WH, et al. Cost-Effectiveness of Endovascular Stroke Therapy: A Patient Subgroup Analysis From a US Healthcare Perspective. *Stroke* 2016; 47: 2797–2804.

12. Peultier A-C, Pandya A, Sharma R, et al. Cost-effectiveness of Mechanical Thrombectomy More Than 6 Hours After Symptom Onset Among Patients With Acute Ischemic Stroke. *JAMA Network Open* 2020; 3: e2012476.

13. Nguyen CP, Lahr MM, van der Zee D-J, et al. Cost-effectiveness of tenecteplase versus alteplase for acute ischemic stroke. *Eur Stroke J* 2023; 8: 638–646.

14. Nguyen CP, Lahr MMH, Zee D-J van der, et al. Cost-effectiveness of Direct Transfer to Angiography Suite of Patients With Suspected Large Vessel Occlusion. *Neurology* 2023; 101: e1036–e1045.

15. Jackson C, Heath A, package) GB(Author of code taken from the B, et al. Voi: Expected Value of Information, https://cran.r-project.org/web/packages/voi/index.html (2024, accessed 6 February 2025).

16. Vervaart M. Calculating the Expected Net Benefit of Sampling for Survival Data: A Tutorial and Case Study. *Med Decis Making* 2024; 44: 719–741.

17. Dutch National Health Care Institute. Cost-effectiveness in practice, https://www.zorginstituutne derland.nl/publicaties/rapport/2015/06/26/kosteneffectiviteit-in-de-praktijk (2015, accessed 13 November 2023).

18. Skajaa N, Adelborg K, Horváth-Puhó E, et al. Risks of Stroke Recurrence and Mortality After First and Recurrent Strokes in Denmark: A Nationwide Registry Study. *Neurology* 2022; 98: e329–e342.

19. de Havenon A, Viscoli C, Kleindorfer D, et al. Disability and Recurrent Stroke Among Participants in Stroke Prevention Trials. *JAMA Network Open* 2024; 7: e2423677.

20. Statistics Netherlands (CBS). Life expectancy; gender, age (per year and period of five years), https://opendata.cbs.nl/statline/#/CBS/nl/dataset/37360ned/table?ts=1699004219887 (accessed 3 November 2023).

21. Shavelle RM, Brooks JC, Strauss DJ, et al. Life Expectancy after Stroke Based On Age, Sex, and Rankin Grade of Disability: A Synthesis. *Journal of Stroke and Cerebrovascular Diseases* 2019; 28: 104450.

22. Huybrechts KF, Caro JJ, Xenakis JJ, et al. The Prognostic Value of the Modified Rankin Scale Score for Long-Term Survival after First-Ever Stroke. *Cerebrovasc Dis* 2008; 26: 381–387.

23. Dutch National Health Care Institute. Farmacotherapeutisch Kompas, https://www.farmacotherapeutisch kompas.nl/ (accessed 3 November 2023).

24. Buisman LR, Tan SS, Nederkoorn PJ, et al. Hospital costs of ischemic stroke and TIA in the Netherlands. *Neurology* 2015; 84: 2208–2215.

25. Hakkaart-van Roijen L, Peeters S, Kanters T. *Costing manual: Methods and Reference Prices for Economic Evaluations in Healthcare.* Dutch National Health Care Institute, 2024.

26. van den Berg LA, Berkhemer OA, Fransen PSS, et al. Economic Evaluation of Endovascular Treatment for Acute Ischemic Stroke. *Stroke* 2022; 53: 968–975.

27. National Institute for Health and Care Excellence. Pembrolizumab with axitinib for untreated advanced renal cell carcinoma [TA650], https://www.nice.org.uk/guidance/ta650/ (2020, accessed 17 June 2022).

28. Voorst H van, Kunz WG, Berg LA van den, et al. Quantified health and cost effects of faster endovascular treatment for large vessel ischemic stroke patients in the Netherlands. *Journal of NeuroInterventional Surgery* 2021; 13: 1099–1105.

29. van Baal PHM, Wong A, Slobbe LCJ, et al. Standardizing the Inclusion of Indirect Medical Costs in Economic Evaluations. *Pharmacoeconomics* 2011; 29: 175–187.

30. Buuren S van, Groothuis-Oudshoorn K. Mice: Multivariate Imputation by Chained Equations in R. *Journal of Statistical Software* 2011; 45: 1–67.

31. Barnard J, Rubin DB. Small-Sample Degrees of Freedom with Multiple Imputation. *Biometrika* 1999; 86: 948–955.
